# Surface plasmon resonance of Au-Cu bimetallic nanoparticles predicted by a quasi-chemical model

**DOI:** 10.1186/1556-276X-8-408

**Published:** 2013-10-02

**Authors:** Yen-Hsun Su, Wen-Lin Wang

**Affiliations:** 1 Department of Materials Science and Engineering, National Cheng Kung University, Tainan, Taiwan; 2 Advanced Optoelectronic Technology Center, National Cheng Kung University, Tainan, Taiwan

**Keywords:** Dielectric properties, Gibbs free energy, Nanostructures, Metals, Optical properties

## Abstract

Au-Cu alloys are functional materials with nonlinear optical applications. However, the optical properties of such alloys are difficult to predict due to the random mixing of materials. In this paper, we present a quasi-chemical model to simulate the optical properties of Au-Cu alloy systems based on the mixing of Gibbs free energy. This model is also able to predict the position of the surface plasmon resonance peaks for Au-Cu alloy nanoparticles. The model can be applied to predict the optical properties of alloy systems in the fields of plasmonics and nanophotonics.

## Background

Metal nanoparticles (NPs) have attracted much research interest due to their unusual chemical and physical properties, such as catalytic activity, novel electronics, optics, and magnetic properties, and they have potential applications in solar cells and biosensors [1-7].

Alloy nanoparticle systems have been found to exhibit optical limiting properties due to surface plasmon resonance and have been used in biodiagnostic applications [8,9]. Alloy nanoparticles are materials used to tune the position of surface plasmon resonance, and thus help to produce materials for use in nonlinear optical applications [10-14]. Au-Cu alloy system is a completely dissoluble alloy. The position of surface plasmon resonance for Au NPs is about 520 nm. The position of surface plasmon resonance for Cu NPs is 570 ~ 580 nm [15]. At low temperatures, Au, Au_3_Cu, AuCu, AuCu_3_, and Cu exist and order easily in Au-Cu alloys system. The prediction of the optical properties of such alloy systems is desirable if they are to be used in the design of optical devices. However, the optical properties of alloy systems are difficult to predict because of the random mixing of materials.

The quasi-chemical method is a statistical approach for predicting the short-range-order of Au-Cu alloys system according to Gibbs free energy. While the optical properties of Au-Cu alloys can be computed by the quasi-chemical model based on the energy potential between the electric field and induced dipole, few works have attempted to do this.

In this study, we thus simulate the optical properties of Au and Cu using a quasi-chemical model, based on the energy potential between the electric field and induced dipole. We then used this quasi-chemical method to modify the statistics for the short-range-order of Au-Cu alloy system. Then the optical properties are simulated by combining the Gibbs free energy and electric potential energy. The light extinction of nanoparticles is calculated by using Mie theory. The results show that the model is suitable for predicting the position of surface plasmon resonance peaks.

## Methods

### Model

#### Regular solution

Au-Cu alloy system refers to a solid solution. Properties of a regular solution are best examined based on the concept of excess function [16]. The excess value of an extensive thermodynamic solution property is simply the difference between its actual value and the value that it would have if the solution were ideal based on the Gibbs free energy of the solution,

(1) $$G=G^{\mathrm{id}}+G^{\mathrm{XS}}$$

In which *G* is the molar Gibbs free energy of the solution, *G*^id^ is the molar Gibbs free energy that the solution would have if it were ideal, and *G*^XS^ is the excess molar Gibbs free energy of the solution. Because the two components have equal molar volumes and do not exhibit a change in molar volume when mixed, their regular solution behavior can be understood by the application of a statistical mixing model, i.e., a quasi-chemical model.

#### Quasi-chemical model

The energy of the solution is the sum of its interatomic bond energies. Consider 1 mol of a mixed crystal containing N_A_ atoms of A and N_B_ atoms of B such that

(2) $$X_{A}=\frac{N_{A}}{N_{A}+N_{B}}=\frac{N_{A}}{N_{O}}\;\mathrm{and}\;X_{B}=\frac{N_{B}}{N_{O}},$$

where *N*_O_ is Avogadro’s number. The mixed crystal, or solid solution, contains three types of atomic bond: A-A bonds, B-B bonds, and A-B bonds. A-A bonds the energy of each of which is U_AA_, B-B bonds the energy of each of which is U_BB_, A-B bonds the energy of each of which is U_AB_. If in the solution, there are P_AA_ A-A bonds, P_BB_ B-B bonds, and P_AB_ A-B bonds, the energy of the solution *U* is obtained as the linear combination

(3) $$U=P_{\text{AA}}U_{\text{AA}}+P_{\text{BB}}U_{\text{BB}}+P_{\text{AB}}U_{\text{AB}}$$

and the problem of calculating *U* becomes one of calculating the values of *P*_AA_, *P*_BB_, and *P*_AB_. Thus,

(4) $$\Delta U^{M}=P_{\text{AB}}\left[U_{\text{AB}}-\frac{1}{2}\left(U_{\text{AA}}+U_{\text{BB}}\right)\right].$$

The change in volume is negligible. Since *ΔV*^M^ = 0,

(5) $$\Delta H^{M}=\Delta U^{M}=P_{\text{AB}}\left[U_{\text{AB}}-\frac{1}{2}\left(U_{\text{AA}}+U_{\text{BB}}\right)\right].$$

Ideal mixing requires the condition *U*_AB_ = *U*_AA_ = *U*_BB_. If *ΔH*^M^ = 0, the mixing of the N_A_ atoms with the N_B_ atoms of B is random.

(6) $$\Delta S^{M}=\Delta S^{M,\mathrm{id}}=-R\left(X_{A}\ln X_{A}+X_{B}\ln X_{B}\right).$$

The quasi-chemical model is a statistical mixing model in Gibbs free energy. According to Equations 5 and 6, the mixing Gibbs free energy will be presented. In the ‘Results and discussion’ section, the dipole energy in Gibbs free energy was utilized to consider the optical properties with different frequencies of incident light.

## Results and discussion

The probability that a neighboring pair of sites contains an A-B pair is 2*X*_A_*X*_B_, an A-A pair is *X*_A_^2^, and B-B pair is *X*_B_^2^, and $X_{A}^{2}+2X_{A}X_{B}+X_{B}^{2}={\left(X_{A}+X_{B}\right)}^{2}=1$ The quasi-chemical model is a statistical mixing model that describes the mixing cluster. The difference in Gibbs energy is presented as follows:

(7) $$\Delta G^{M}=\Delta H^{M}-T\Delta S^{M}.$$

Combining Equations 5 and 6 with Equation 7 produces the following:

(8) $$\begin{array}{l} \Delta G^{M}=P_{\mathrm{AB}}\left[U_{\mathrm{AB}}-\frac{1}{2}\left(U_{\mathrm{AA}}+U_{\mathrm{BB}}\right)\right]\\ \;+\mathrm{RT}\left(X_{A}\ln X_{A}+X_{B}\ln X_{B}\right). \end{array}$$

Because *P*_AB_ = 2*X*_A_*X*_B_,

(9) $$\begin{array}{l} \Delta G^{M}=2X_{A}X_{B}\left[U_{\mathrm{AB}}-\frac{1}{2}\left(U_{\mathrm{AA}}+U_{\mathrm{BB}}\right)\right]\\ \;+\mathrm{RT}\left(X_{A}\ln X_{A}+X_{B}\ln X_{B}\right). \end{array}$$

The Gibbs free energy of the solution is as follows:

(10) $$\begin{array}{l} G=G^{0}+\Delta G^{M}=X_{A}{}^{2}G_{A}^{0}+X_{B}{}^{2}G_{B}^{0}\\ \;+2X_{A}X_{B}\left[U_{\mathrm{AB}}-\frac{1}{2}\left(U_{\mathrm{AA}}+U_{\mathrm{BB}}\right)\right]\\ \;+\mathrm{RT}\left(X_{A}\ln X_{A}+X_{B}\ln X_{B}\right). \end{array}$$

After applying the electric field $\vec{E}$,

(11) $$\begin{array}{l} G-{\vec{p}}_{\mathrm{mixing}}\cdot \vec{E}=X_{A}{}^{2}\left(G_{A}^{0}-{\vec{p}}_{A}\cdot \vec{E}\right)\\ \;+X_{B}{}^{2}\left(G_{B}^{0}-{\vec{p}}_{B}\cdot \vec{E}\right)\\ \;+2X_{A}X_{B}[\left(U_{\mathrm{AB}}-{\vec{p}}_{\mathrm{AB}}\cdot \vec{E}\right)-\frac{1}{2}(\left(U_{\mathrm{AA}}-{\vec{p}}_{A}\cdot \vec{E}\right)\\ \;+\left(U_{\mathrm{BB}}-{\vec{p}}_{B}\cdot \vec{E}\right))]\\ \;+\mathrm{RT}\left(X_{A}\ln X_{A}+X_{B}\ln X_{B}\right). \end{array}$$

where ${\vec{p}}_{\mathrm{mixing}}$ is the induced dipole moment of metamaterial, ${\vec{p}}_{A}$ is the induced dipole moment of material A, ${\vec{p}}_{B}$ is the induced dipole moment of material B, and ${\vec{p}}_{\mathrm{AB}}$ is the induced dipole moment due to the interaction of materials A and B.

The Gibbs energy was subtracted when applying an electric field from that without applying one, as follows:

(12) $$\begin{array}{l} {\vec{p}}_{\mathrm{mixing}}\cdot \vec{E}=X_{A}{}^{2}\left({\vec{p}}_{A}\cdot \vec{E}\right)+X_{B}{}^{2}\left({\vec{p}}_{B}\cdot \vec{E}\right)\\ \;+2X_{A}X_{B}\left[\left({\vec{p}}_{\mathrm{AB}}\cdot \vec{E}\right)-\frac{1}{2}\left(\left({\vec{p}}_{A}\cdot \vec{E}\right)+\left({\vec{p}}_{B}\cdot \vec{E}\right)\right)\right]. \end{array}$$

Because $\vec{p}=\left(\epsilon -\epsilon_{0}\right)\vec{E}$, the above equation can be rewritten as follows:

(13) $$\begin{array}{l} \left(\epsilon_{\mathrm{mixing}}-\epsilon_{0}\right)\vec{E}\cdot \vec{E}=X_{A}{}^{2}\left(\left(\epsilon_{A}-\epsilon_{0}\right)\vec{E}\cdot \vec{E}\right)\\ \;+X_{B}{}^{2}\left(\left(\epsilon_{B}-\epsilon_{0}\right)\vec{E}\cdot \vec{E}\right)\\ \;+2X_{A}X_{B}[\left(\left(\epsilon_{\mathrm{AB}}-\epsilon_{0}\right)\vec{E}\cdot \vec{E}\right)-\frac{1}{2}(\left(\left(\epsilon_{A}-\epsilon_{0}\right)\vec{E}\cdot \vec{E}\right)\\ \;+\left(\left(\epsilon_{B}-\epsilon_{0}\right)\vec{E}\cdot \vec{E}\right))] \end{array}$$

(14) $$\begin{array}{l} \left(\epsilon_{\mathrm{mixing}}-\epsilon_{0}\right)=X_{A}{}^{2}\left(\epsilon_{A}-\epsilon_{0}\right)+X_{B}{}^{2}\left(\epsilon_{B}-\epsilon_{0}\right)\\ \;+2X_{A}X_{B}\left[\left(\epsilon_{\mathrm{AB}}-\epsilon_{0}\right)-\frac{1}{2}\left(\left(\epsilon_{A}-\epsilon_{0}\right)+\left(\epsilon_{B}-\epsilon_{0}\right)\right)\right]\\ \left(\epsilon_{\mathrm{mixing}}-\epsilon_{0}\right)=X_{A}{}^{2}\left(\epsilon_{A}-\epsilon_{0}\right)\\ \;+X_{B}{}^{2}\left(\epsilon_{B}-\epsilon_{0}\right)+2X_{A}X_{B}\left[\left(\epsilon_{\mathrm{AB}}-\frac{1}{2}\left(\epsilon_{A}+\epsilon_{B}\right)\right)\right]\\ \epsilon_{\mathrm{mixing}}=\epsilon_{A}X_{A}{}^{2}+\epsilon_{B}X_{B}{}^{2}+2X_{A}X_{B}\left[\left(\epsilon_{\mathrm{AB}}-\frac{1}{2}\left(\epsilon_{A}+\epsilon_{B}\right)-\epsilon_{0}\right)\right]. \end{array}$$

The dielectric function of the mixed material includes the interaction term $\left[(\epsilon_{\mathrm{AB}}-\frac{1}{2}\left(\epsilon_{A}+\epsilon_{B}\right)-\epsilon_{0}\right]$ and independent terms *ϵ*_A_*X*_A_^2^ and *ϵ*_B_*X*_B_^2^. When $\left[(\epsilon_{\mathrm{AB}}-\frac{1}{2}\left(\epsilon_{A}+\epsilon_{B}\right)-\epsilon_{0}\right]$ is assumed to be an experience constant, *Λ*, the dielectric function of mixing material is reduced to the following form:

(15) $$\epsilon_{\mathrm{mixing}}=\epsilon_{A}X_{A}{}^{2}+\epsilon_{B}X_{B}{}^{2}+2X_{A}X_{B}\Lambda$$

The Newton formula [17] is used to apply these concepts to the clustered material. The dielectric functions refer to these clusters and the embedding matrix,

(16) $$\epsilon_{\mathrm{Newton}‒\mathrm{mixing}}=f\epsilon_{A}+\left(1-f\right)\epsilon_{B}.$$

The quasi-chemical model describes the mixing of clusters in which the interaction term is approximated by Newton formula mixing. Combining the probability of neighboring pairs with the Newton formula, the optical model of the regular solution is as follows:

(17) $$\epsilon_{\text{eff}}=X_{A}^{2}\epsilon_{A}+2X_{A}X_{B}\left(\epsilon_{\text{Newton}‒\text{mixing}}\right)+X_{B}^{2}\epsilon_{B}.$$

**Figure 1:**
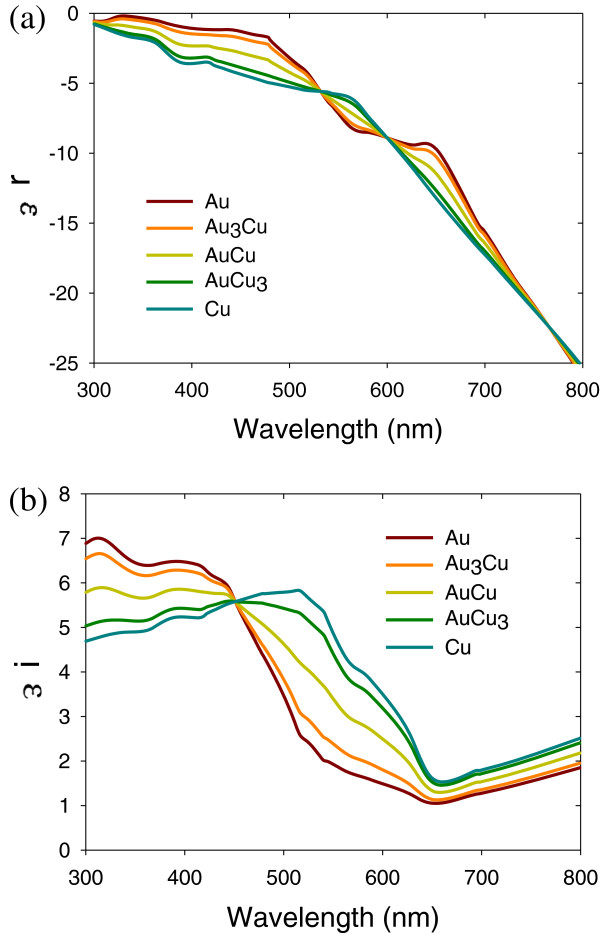
**Effective dielectric complex of the alloy. (a)** Real part, *ϵ*_r_. **(b)** Imaginary part, *ϵ*_i_, of the dielectric complex of Au-Cu alloy.

The effective dielectric complex of the alloy is presented in Figure 1.

**Figure 2:**
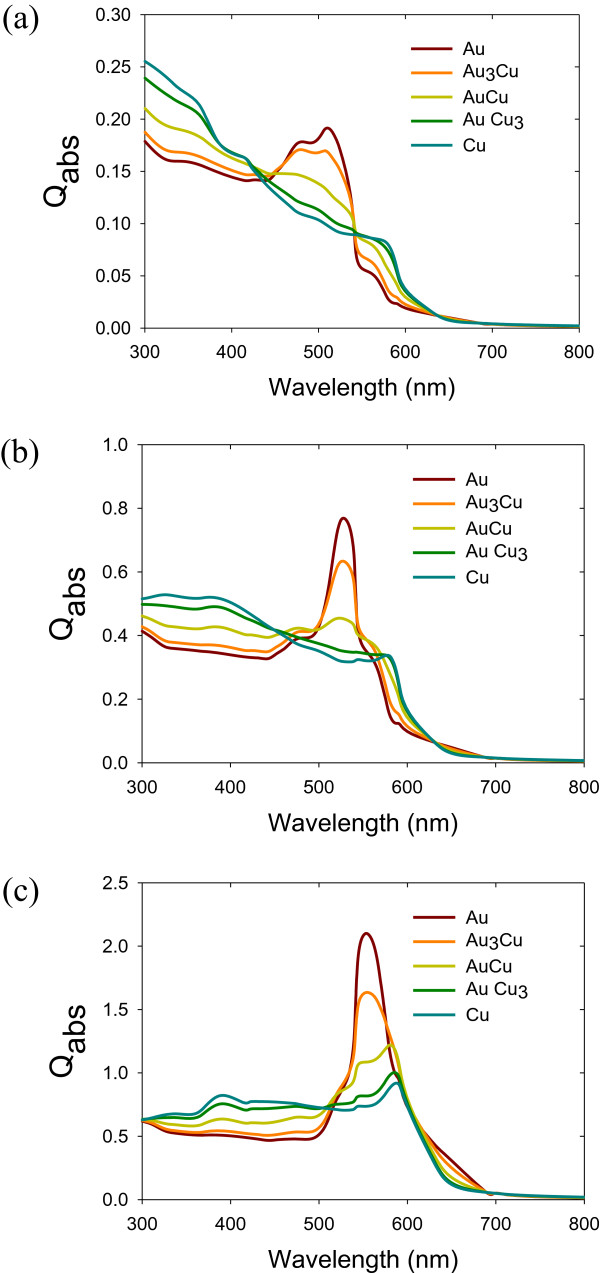
**Extinction of Au-Cu alloy nanoparticles.** Extinction of Au-Cu alloy nanoparticles (10 nm) when **(a)***n* = 1, **(b)***n* = 1.4, and **(c)***n* = 1.8 (*Q*_abs_ is the extinction coefficient).

**Figure 3:**
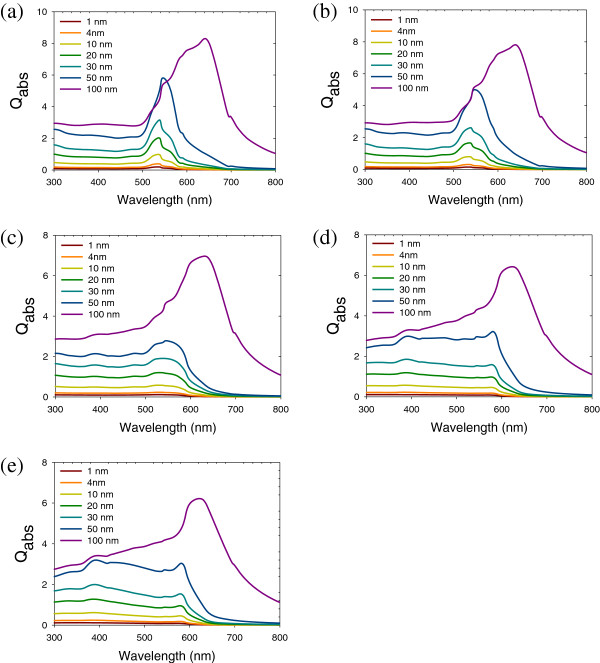
**Extinction of different sized NPs. (a)** Au, **(b)** Au_3_Cu, **(c)** AuCu, **(d)** AuCu_3_, and **(e)** Cu alloy nanoparticles (*n* = 1; *Q*_abs_ is the extinction coefficient).

**Figure 4:**
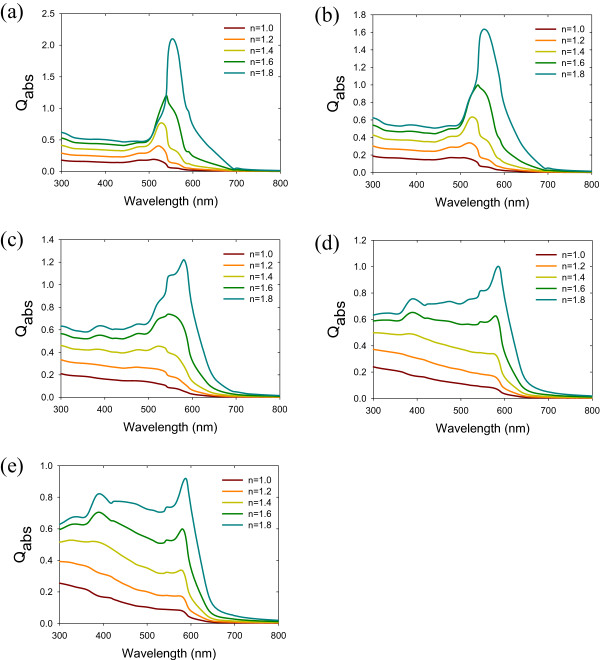
**Extinction of different refractive index. (a)** Au, **(b)** Au_3_Cu, **(c)** AuCu, **(d)** AuCu_3_, and **(e)** Cu alloy nanoparticles.

According to Mie theory [18,19], the resonances denoted as surface plasmon were relative with the onset of the quantum size and shape effects of Au NPs. There is one SPR band for metal NPs, and this is shown as follows [20,21]:

(18) $$\epsilon_{i}=\epsilon_{h}+f\frac{\epsilon_{h}\left(\epsilon_{h}-\epsilon_{m}\right)}{\Gamma_{i}\epsilon_{m}+\left(1-\Gamma_{i}\right)\epsilon_{h}},$$

where *ϵ*_h_ is the dielectric constant of the host medium embedding Au NPs, *ϵ*_m_ is the dielectric constant of Au NPs, *f* is the volume fraction of Au NPs, *ϵ*_i_ is the total dielectric constant, and Γ_*i*_ is a set of three parameters defined along the principal axes of the particle characterizing its shape. Γ_1_ + Γ_2_ + Γ_3_ = 1 and the other parameters range from 0 to 1. The frequencies of the surface plasmon of nonspherical metal NPs have two or three bands, depending on their shape. The extinction coefficients of alloy metal NPs with different sizes and environments are presented in Figures 2, 3, 4.

The quasi-chemical model is used to calculate the optical properties of Au-Cu alloys. The real part of the dielectric complex is negative for Au-Cu alloy system. The imaginary part of dielectric constant for Au-Cu alloy system shows the peaks that appear in range from 430 to 520 nm due to the electronic transition between the d band and sp band. The real and imaginary parts of the dielectric complex for Au-Cu alloys system are as shown in Figure 1a,b, respectively.

We use Mie theory to predict the spectrum and position of surface plasmon resonance. Figure 2b shows the extinction of a 10-nm diameter Au-Cu nanoparticle in different refractive index surroundings. For *n* = 1.4, the surface plasmon resonance peaks are 532, 538, 561, 567, and 578 nm for Au, Au_3_Cu, AuCu, AuCu_3_, and Cu, respectively, and these results which are in agreement with those of other experimental results [22].

The extinction spectra of Au-Cu bimetallic nanoparticle with size effect are presented in Figure 3. As the size of nanoparticles increase, the peak of surface plasmon resonance red-shifts. When the size is less than 50 nm, the size effect becomes more significant. The higher the ratio of Cu to Au of is, the more the surface plasmon resonance red-shifts. When the size is greater than 50 nm, the size effect is less significant due to the small increase in the cross section. The refractive index effect is shown in Figure 4. As the refractive index increases, the surface resonance peak will red-shift and become increasingly sharp. Based on this, it is possible to predict the surface plasmon resonance peaks of regular solution alloys, such as Au-Cu, Cu-Ag, Ag-Cu, and Au-Cu-Ag systems.

## Conclusion

In this work we used the quasi-chemical model to compute the optical properties of Au-Cu alloy system. The results show that it is possible to use this approach to predict the positions of surface plasmon resonance peaks. This model is thus a useful tool in the development of for future applications of alloy nanoparticles for plasmonics and nanophotonics.

## Competing interests

The authors declare that they have no competing interests.

## Authors’ contributions

YHS and WLW contribute in writing and model setting in all these works. Both authors read and approved the final manuscript.

## Authors’ information

YHS is an assistant professor and WLW is a student in the Department of Materials Science and Engineering in National Cheng Kung University, Taiwan.
